# Predicting the 2-Year Risk of Progression from Prediabetes to Diabetes Using Machine Learning among Chinese Elderly Adults

**DOI:** 10.3390/jpm12071055

**Published:** 2022-06-27

**Authors:** Qing Liu, Qing Zhou, Yifeng He, Jingui Zou, Yan Guo, Yaqiong Yan

**Affiliations:** 1 Department of Epidemiology, School of Public Health, Wuhan University, Wuhan 430071, China; liuqing@whu.edu.cn (Q.L.); zhouqing@whu.edu.cn (Q.Z.); 2 School of Geodesy and Geomatics, Wuhan University, Wuhan 430079, China; heyifeng@whu.edu.cn (Y.H.); jgzou@sgg.whu.edu.cn (J.Z.); 3 Wuhan Center for Disease Control and Prevention, Wuhan 430015, China; swallow315@whcdc.org

**Keywords:** machine learning, prediabetes, incident diabetes, predictive models

## Abstract

Identifying people with a high risk of developing diabetes among those with prediabetes may facilitate the implementation of a targeted lifestyle and pharmacological interventions. We aimed to establish machine learning models based on demographic and clinical characteristics to predict the risk of incident diabetes. We used data from the free medical examination service project for elderly people who were 65 years or older to develop logistic regression (LR), decision tree (DT), random forest (RF), and extreme gradient boosting (XGBoost) machine learning models for the follow-up results of 2019 and 2020 and performed internal validation. The receiver operating characteristic (ROC), sensitivity, specificity, accuracy, and F1 score were used to select the model with better performance. The average annual progression rate to diabetes in prediabetic elderly people was 14.21%. Each model was trained using eight features and one outcome variable from 9607 prediabetic individuals, and the performance of the models was assessed in 2402 prediabetes patients. The predictive ability of four models in the first year was better than in the second year. The XGBoost model performed relatively efficiently (ROC: 0.6742 for 2019 and 0.6707 for 2020). We established and compared four machine learning models to predict the risk of progression from prediabetes to diabetes. Although there was little difference in the performance of the four models, the XGBoost model had a relatively good ROC value, which might perform well in future exploration in this field.

## 1. Introduction

Diabetes is one of the significant public problems worldwide, resulting in 536.6 million adults with diabetes, 541.0 million adults with impaired glucose tolerance (IGT), and 319.0 million adults with impaired fasting glucose (IFG) [1]. Prediabetes is often used to refer to the latter two states and is more commonly observed in the elderly [2]. Due to the growing economic burden and mortality caused by diabetes, the prevention of diabetes is imminent. Unlike incurable diabetes, the majority of prediabetes patients, especially the elderly, may revert to normoglycaemia or remain stable. Only a fraction of patients with prediabetes progress to diabetes [3], and this proportion can be further reduced by lifestyle and pharmacological interventions [4]. So, identifying people with a high risk of developing diabetes among prediabetic patients may facilitate the implementation of targeted interventions and avoid the burden of prevention for people at low risk. 

Machine learning has been identified as a powerful tool for application in the medical field [5]. According to electronic health records, Neves et al. [6] predicted the outcome of diabetes by applying Bayesian Networks. Lama et al. [7] used a random forest (RF) classifier to train a model for predicting whether an individual develops prediabetes or type 2 diabetes. Meng et al. [8] developed three multiple prediction models with logistic regression (LR), artificial neural networks, and decision tree (DT) for predicting diabetes or prediabetes. However, most machine learning models in the field of diabetes research are aimed at the onset and complications. Prediction models of progression from prediabetes to diabetes are limited, and they may not be reliable to generalize to Chinese people due to ethnicity differences [9].

Thus, the purpose of this study is to train machine learning models for predicting patients with prediabetes progress to diabetes based on demographic information and laboratory results. We select LR, DT, RF, and extreme gradient boosting (XGBoost) to build predictive models and optimize their hyperparameters by 10-fold cross-validation. Accuracy, sensitivity, specificity, and receiver operating characteristic (ROC) are also used to estimate the performance of these predictive models.

## 2. Materials and Methods

### 2.1. Study Design and Participants

We conducted a retrospective cohort study of participants who attended free health screening service in Wuhan, China, between 2018 and 2020. This project has provided annual physical examinations to adults older than 65 years, which covered 31.3% of the elderly population in Wuhan (388,420/1,242,470, in 2018).

**Figure 1:**
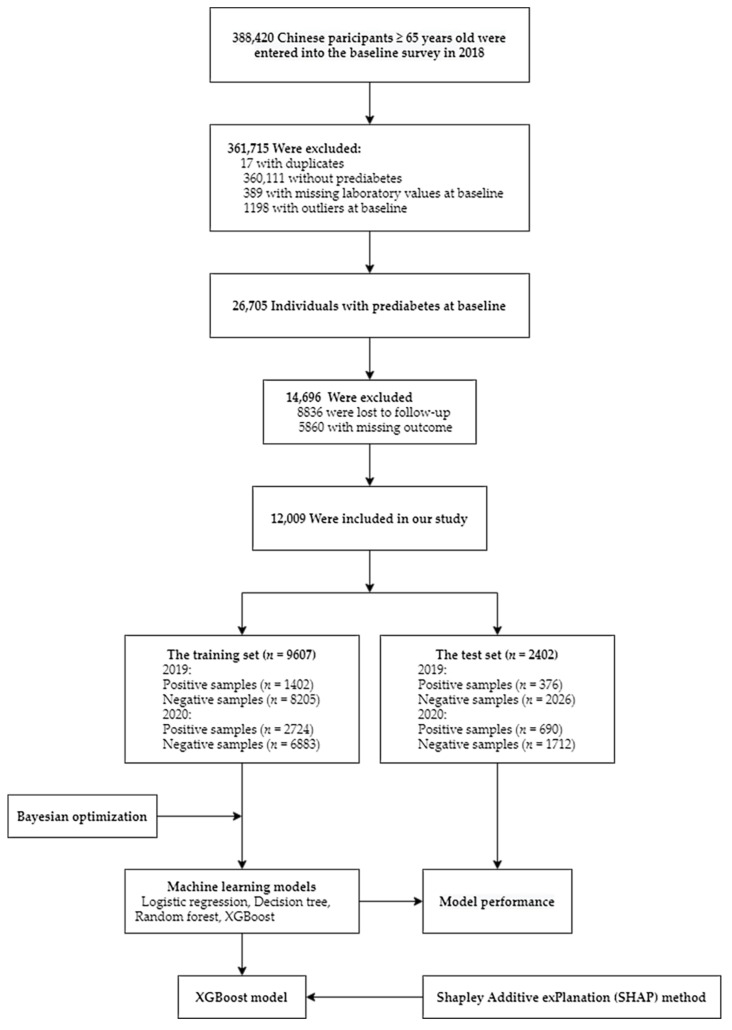
Flowchart of study participants.

We restricted our study to 26705 participants with prediabetes at baseline whose fasting plasma glucose (FPG) ≥ 6.1 mmol/L [10] and did not meet the criteria of diabetes as defined below. Those who had missing outcomes or were lost to follow-up were excluded (Figure 1). Available data included demographics, lifestyle, medical history, anthropometric indices, and laboratory results. Ethical approval was obtained from the Ethics Committee of Wuhan Center for Disease Control and Prevention (#WHCDCIRB-K-2018023). 

### 2.2. Data Collection

Demographic characteristics included age, gender, marital status, and education level. Lifestyle included smoking, drinking, and exercise. An anthropometric examination was conducted by well-trained community physicians. Height and weight were measured with subjects wearing light clothes without shoes. The body mass index (BMI) was calculated as the individual’s body weight (kg) divided by the square of height (m). Waist circumference (WC) was measured at the midpoint between the last rib and iliac crest. Blood pressure was measured three times by an electronic sphygmomanometer when participants were in a sitting position after 5 minutes of rest. Blood samples were drawn from individuals after at least 8 hours of fasting for laboratory tests. Exercise was defined as those who had more than three times of physical activity for 30 min per week. Smoking was defined as those who reported smoking at least once per month. Drinking was defined as those who drink alcohol more than once a month. 

### 2.3. Definition of Outcome

An individual was regarded to reach the outcome of diabetes when FPG ≥ 7.0 mmol/L according to the American Diabetes Association diagnostic criteria [11] or a self-reported diagnosis by health care professionals during the follow-up.

### 2.4. Feature Selection

To reduce the computational complexity and generalization error of the model, it was important to determine which variables were most relevant. We selected the least absolute shrinkage and selection operator (LASSO) regression analysis to screen the candidate features. Finally, 8 features that included education, BMI, WC, FPG, total cholesterol (TC), triglyceride (TG), high density lipoprotein cholesterol (HDL-C), and Alanine aminotransferase (ALT) were selected to develop a machine learning model.

### 2.5. Machine Learning Model Development and Evaluation

The processed data were randomly divided into a training set and a test set in a 4:1 ratio. In order to explore the differences in predictive ability and risk factors between 1-year and 2-year risk of diabetes onset, we constructed machine learning models for two forecast periods. Four machine learning algorithms, including LR, DT, RF, and XGBoost were used to develop models on the training set. LR is a linear model for classification, which predicts a probability value of occurrence of the objective using a sigmoid function and is widely used in biomedicine [12]. A decision tree is a flowchart-like tree structure, where each attribute can represent one internal node in a generated decision tree and has as many branches as its number of different value classes. Moreover, the final leaves of a decision tree represent the decision attribute [13]. Random forest is a supervised learning algorithm that randomly extracts multiple samples from the training set using a bootstrap algorithm and then generates multiple decision trees [14]. The classification results of new instances are determined by taking a majority vote over all the decision trees. XGBoost is an ensemble machine learning algorithm based on decision tree, which was first proposed by Chen and Guestrin [15]. As an optimized implementation of gradient boosting [16], XGBoost shows excellent performance in regression and classification tasks. 

Hyperparameters of each model are important for model performance. We performed a 10-fold cross-validation for automated Bayesian optimization with 500 iterations to obtain optimized hyperparameters of each model.

All the machine learning models were assessed for their risk discrimination performance ROC curves on the test set. Multiple indicators containing sensitivity, specificity, accuracy, and F1 score were used to evaluate the predictive ability of four models. We further applied the Shapley Additive exPlanation (SHAP) algorithm to the training set for the model explanation.

### 2.6. Statistical Analysis

Analysis of statistical description was performed by SAS (version 9.4). Data were expressed as means ± standard deviation (normally distributed) or median (interquartile range) (non-normally distributed). Categorical variables were shown as frequency and percentages. A comparison among groups was conducted by one-way ANOVA, Wilcoxon rank-sum test, or Chi-square test according to the data types. P values were two-tailed and were considered to be significant when they were < 0.05. All model development and optimization were achieved by Python (version 3.11).

## 3. Results

### 3.1. Baseline Characteristics of Data Sets Used for the Analysis

The baseline characteristics between the groups of participants with incident diabetes at different time points are presented in Table 1. Within the free health screening project, 12009 elderly prediabetic subjects who met the inclusion criteria were included in our study. All the participants had complete information on demographics, lifestyles, medical history, and laboratory tests. During the two-year follow-up, a total of 3414 individuals progressed to diabetes from prediabetes, and their average annual rate of diabetes progression was 14.21%. 

At baseline, the majority of the study population had primary school and lower education levels. The distribution of education was shown in the following categories: primary school and lower: 7456 (62.09%); middle school: 2424 (20.18%); high school: 1134 (9.44%); and university and higher: 995 (8.29%). The mean BMI was 24.69 ± 3.42 kg/m^2^. The mean WC was 86.02 ± 9.72 cm. The mean FPG was 6.44 ± 0.25 mmol/L. The mean TC was 5.04 ± 1.05 mmol/L. The median TG was 1.34 (0.97). The mean HDL-C was 1.38 ± 0.42 mmol/L, and the median ALT was 18.20 (11.00). 

### 3.2. Performance Comparison between Different Machine Learning Models

Four different machine learning models using LR, DT, RF, and XGBoost were constructed for two forecast periods: 1 and 2 years.

#### 3.2.1. 1-Year Forecast Period

**Figure 2:**
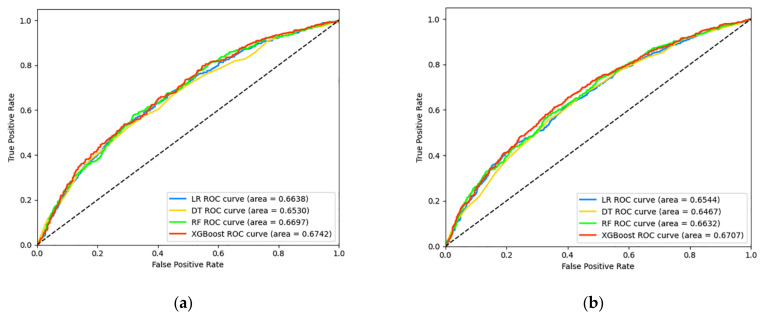
Receiver operating characteristic (ROC) curves derived for prediction horizon of 1 and 2 years using the four models based logistic regression (LR), decision tree (DT), random forest (RF), and extreme gradient boosting (XGBoost): (**a**) 1-year forecast period; (**b**) 2-year forecast period.

**Figure 3:**
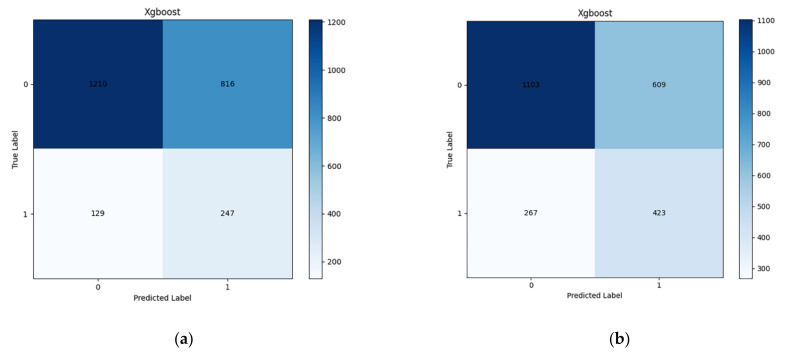
Confusion matrices derived for prediction horizons of 1 and 2 years based on the extreme gradient boosting (XGBoost): (**a**) 1-year forecast period; (**b**) 2-year forecast period.

Among these 12009 participants, 1778 (14.81%) had developed diabetes within 1 year after baseline. The performance of the four machine learning models is displayed in Figure 2a and Table 2. All the models obtained the optimal hyperparameters using Bayesian optimization except LR (with default hyperparameters). The XGBoost model performed relatively well (ROC: 0.6742), followed by the RF model (ROC: 0.6697), and the DT model ranked last (ROC: 0.6530). Due to the imbalance ratio reaching 5.75, we identified the optimal threshold using an ROC curve. The XGBoost model showed good sensitivity (0.6569) but relatively poor specificity (0.5972) and accuracy (0.6066). The F1 score of XGBoost ranked second among these models. The confusion matrix of XGBoost is presented in Figure 3a.

#### 3.2.2. 2-Year Forecast Period

The number of incident diabetes reached 3414 (28.43%) during the 2-year follow-up. The performance of the four machine learning models is presented in Figure 2b and Table 2. The ROC value of all models for the 2-year forecast period was lower than for the 1-year forecast period. The XGBoost model still performed relatively efficiently, with a comparatively higher ROC value of 0.6707. The threshold was adjusted again because of an increased number of positive cases. The imbalance ratio decreased to 2.52, and the model for predicting 2-year risk changed accordingly. The optimal threshold was inferred by the ROC curve and increased from 0.14 (1-year forecast period) to 0.30 (2-year forecast period). Compared to the 1-year forecast period, the sensitivity of the XGBoost model decreased, and the specificity and accuracy of XGBoost increased. The F1 score rose to first. The confusion metrix of XGBoost was presented in Figure 3b.

### 3.3. Analysis of Feature Importance

**Figure 4:**
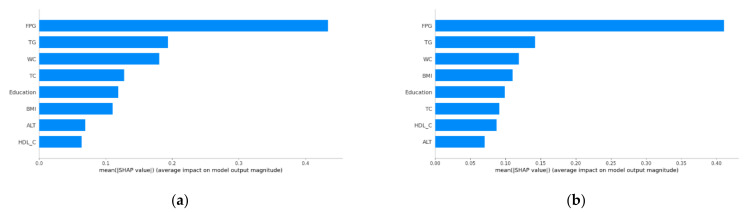
Feature importance in predicting incident diabetes according to the XGBoost model. The Shapley additive explanation (SHAP) algorithm is used to calculate the SHAP value which approximates how much each feature contributes to the average prediction for the dataset. (**a**) 1-year forecast period. (**b**) 2-year forecast period.

**Figure 5:**
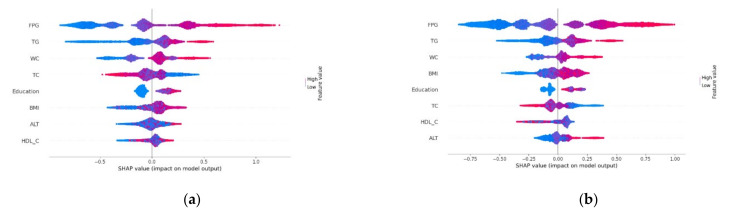
SHAP summary plot of the XGBoost model. (**a**) 1-year forecast period. (**b**) 2-year forecast period.

Taking the XGBoost model with a little higher ROC value (in both forecast periods) and F1 score (in 2-year period) into account, we decided to explain the results of our work based on this machine learning model. To interpret the importance of each feature in the XGBoost model, the ranking of the input features’ importance is shown in Figure 4, and the SHAP summary plot is presented in Figure 5. For two different prediction horizons, FPG, TG, and WC ranked consistently among the top three (Figure 4). The SHAP values of most features decreased to some extent during the 2-year forecast period. In view of the fact that Figure 4 can only show the correlation but not the direction of features, Figure 5 could be a good supplement. The red dots in the SHAP summary plot indicated higher feature values, and the blue dots indicated lower feature values. When the SHAP value of features was greater than zero, such as FPG, TG, WC, BMI, and ALT, that suggested that they were risk factors for diabetes onset.

## 4. Discussion

In this retrospective cohort study, we established and evaluated prediction models for identifying individuals at high risk of progression from prediabetes to diabetes within 1–2 years. The XGBoost model incorporated education, BMI, WC, FPG, TC, TG, HDL-C, and ALT and provided a relatively good classification of risk among all the models overall. However, the discriminatory ability of all models decreased as the forecast period increased. In addition, it was found that there was not much difference in performance among the four models.

In both forecast periods, the XGBoost model performed relatively well. This was not unexpected; the predictive ability of XGBoost has manifested in previous studies of diabetes onset [17] and complications [18]. As an ensemble machine learning algorithm, XGBoost was not affected by the correlation of independent variables, which was exactly the problem that the LR model needed to solve. So, it might be a good choice to use the XGBoost algorithm for modeling in future studies.

Unsurprisingly, consistent with other studies [19,20,21], FPG was the strongest contributor to the models. We also found that the contribution of WC was higher than that of BMI in both forecast periods, which modestly supports the view that the reliability of BMI for determining obesity, a well-known major risk factor for diabetes, was questioned [22] because BMI did not distinguish fat mass from lean mass [23] and WC represented central obesity.

Notably, the proportion of biomarkers reached 62.5% (5/8) among the features included in the models. This confirmed the finding that risk evaluation constructed based on biomarkers was superior to that based on non-laboratory indicators [24]. The inclusion of biomarkers as input in the machine learning modeling will be a trend in the future.

We acknowledge that the performance of our models was not competitive with results presented in the literature for other relative machine learning research [25,26,27]. This may, in part, be attributed to the fact that all of the participants were elderly, who generally had several comorbid diseases. The well-known risk factors and biomarkers in elderly individuals were less sensitive to diabetes onset than in younger adults.

The insufficiency of features might also be one of the reasons why our XGBoost model did not perform as well as in other research [28], whose model included 300 features. After all, in addition to demographic and lifestyle, nutrition intake has also been found to be an important predictor of incident diabetes [29]. However, high-dimensional features generally bring about information redundancy and overfitting problem. Considering that our model included only eight features, we thought this level of performance was acceptable.

Even so, to the best of our knowledge, this was the first study to establish models designed for the prediabetes population in mainland China. The majority of previous studies [30,31,32] focused on the diabetes onset of the general population, ignoring the transitional and high-risk state for the development of diabetes. Given that the proportion of regression to normal glucose levels was much higher than progression towards diabetes among prediabetes [33], changing the screening objects to prediabetes seemed to be more conducive to allocating health resources. 

China faces significant disease and economic burdens due to diabetes and its complications [34]. Identifying high-risk groups among prediabetic patients using the predictive machine learning model we proposed could reduce the economic burden of diabetes through the implementation of targeted lifestyle and pharmacological interventions, even more so given the fact that our model was applicable to China’s national conditions. Aiming to provide free charge essential health services for all citizens, the central government launched the National Basic Public Health Service Program (BPHS), containing 14 items, of which a vital part was geriatric health services [35]. This implied that under the current health policy, no additional data collection would be needed.

Nevertheless, the present study has some limitations worth noting. The major limitation of the present study is the models’ limited performance, which might be related to suboptimal sample sizes and the fewer features. Considering the particularity of our target population, further research should be undertaken to expand the sample size and explore features that are more sensitive to the geriatric population. Second, the number of incident diabetes might be underestimated, for OGTT was not included in the definition of diabetes. However, it is infeasible to use OGTT during a mass free health screening project due to its relatively expensive cost. Moreover, the data used in the study lacked the features known to be diabetes risk factors such as glycosylated hemoglobin and family history of diabetes. In addition, only participants who can be followed up were included in our study. Meanwhile, because developing models could only be based on the participants who reached the follow-up endpoint, we cannot rule out that death could have led to some selection bias. Therefore, the generalization of the research to the whole geriatric population should be cautious. Furthermore, the lack of information on lifestyle changes during follow-up might confound the predictive ability of baseline features. Finally, all the participants included in our study were Chinese, so the predictive model may not be generalizable to other ethnicities.

## 5. Conclusions

In conclusion, we evaluated the performance of several prediction models using four machine learning algorithms based on the demographic, anthropometric indices, and laboratory results. The XGBoost model might be an effective prediction model, which might perform well in future exploration in this field.

## Figures and Tables

**Table 1:** Baseline characteristics between the groups of participants with incident diabetes at different time points.

Variables	2019		*p* Value	2020		*p* Value
	Without DM (*n* = 10,231)	DM (*n* = 1778)		Without DM (*n* = 8595)	DM (*n* = 3414)	
Age (years)	72.06 ± 5.10	72.17 ± 5.22	0.393	72.08 ± 5.13	72.06 ± 5.10	0.813
Gender, *n* (%)			<0.001			0.018
Male	4536 (83.36)	873 (26.14)		3813 (70.49)	1596 (29.51)	
Female	5695 (86.29)	905 (13.71)		4782 (72.45)	1818 (27.55)	
Education, *n* (%)			<0.001			<0.001
≤Primary school	6485 (86.98)	971 (13.02)		5529 (74.16)	1927 (25.84)	
Middle school	1990 (82.10)	434 (17.90)		1615 (66.63)	809 (33.37)	
High school	931 (82.10)	203 (17.90)		764 (67.37)	370 (32.63)	
≥University	825 (82.91)	170 (17.09)		687 (69.05)	308 (9.02)	
Marital status, *n* (%)			0.383			0.897
Married	7762 (84.96)	1374 (15.04)		6541 (71.60)	2595 (28.40)	
Divorced	57 (87.69)	8 (12.31)		44 (67.69)	21 (32.31)	
Widowed	2331 (85.76)	387 (14.24)		1947 (71.63)	771 (28.37)	
Unmarried	81 (90.00)	9 (10.00)		63 (70.00)	27 (30.00)	
Hypertension, *n* (%)			<0.001			<0.001
No	4893 (87.02)	730 (12.98)		4153 (73.86)	1470 (26.14)	
Yes	5338 (85.39)	1048 (16.41)		4442 (69.56)	1944 (30.44)	
Myocardial infarction, *n* (%)			0.463			0.298
No	10,177 (85.18)	1771 (14.82)		8555 (71.60)	3393 (28.40)	
Yes	54 (88.52)	7 (11.48)		40 (65.57)	21 (34.43)	
Coronary heart disease, *n* (%)			0.841			0.144
No	9632 (85.22)	1670 (14.78)		8106 (71.72)	3196 (28.28)	
Yes	599 (84.72)	108 (15.28)		489 (69.17)	218 (30.83)	
Angina pectoris, *n* (%)			0.828			0.437
No	10,187 (85.19)	1771 (14.81)		8556 (71.55)	3402 (28.45)	
Yes	44 (86.27)	7 (13.73)		39 (76.47)	12 (23.53)	
Fatty liver, *n* (%)			0.315			0.055
No	9979 (85.25)	1727 (14.75)		8393 (71.70)	3313 (28.30)	
Yes	252 (83.17)	51 (16.83)		202 (66.67)	101 (33.33)	
Exercise, *n* (%)			0.587			0.455
No	3942 (85.42)	673 (14.58)		3321 (71.96)	1294 (28.04)	
Yes	6289 (85.06)	1105 (14.94)		5274 (71.33)	2120 (28.67)	
Smoking, *n* (%)			0.705			0.883
No	8804 (85.15)	1536 (14.85)		7403 (71.60)	2937 (28.40)	
Yes	1427 (85.50)	242 (14.50)		1192 (71.42)	477 (28.58)	
Drinking, *n* (%)			0.295			0.212
No	8544 (85.35)	1467 (14.65)		7188 (71.80)	2823 (28.20)	
Yes	1687 (84.43)	311 (15.57)		1407 (70.42)	591 (29.58)	
BMI (kg/m^2^)	24.56 ± 3.41	25.48 ± 3.31	<0.001	24.44 ± 3.42	25.34 ± 3.33	<0.001
WC (cm)	85.57 ± 9.73	88.65 ± 9.23	<0.001	85.20 ± 9.68	88.10 ± 9.52	<0.001
SBP (mmHg)	139.17 ± 19.48	140.30 ± 18.70	0.023	138.96 ± 19.52	140.28 ± 18.96	<0.001
DBP (mmHg)	80.99 ± 11.09	81.57 ± 10.64	0.041	80.86 ± 11.11	81.60 ± 10.79	0.001
FPG (mmol/L)	6.42 ± 0.24	6.54 ± 0.26	<0.001	6.40 ± 0.24	6.52 ± 0.26	<0.001
TC (mmol/L)	5.05 ± 1.05	4.99 ± 1.03	0.021	5.06 ± 1.05	5.00 ± 1.03	0.007
TG (mmol/L)	1.32 (0.96)	1.50 (1.06)	<0.001	1.30 (0.93)	1.48 (1.05)	<0.001
HDL-C (mmol/L)	1.39 ± 0.40	1.34 ± 0.51	<0.001	1.40 ± 0.40	1.33 ± 0.44	<0.001
LDL-C (mmol/L)	2.80 ± 0.92	2.76 ± 1.02	0.147	2.78 ± 0.93	2.81 ± 0.95	0.231
ALT (U/L)	18.00 (11.00)	19.10 (12.00)	<0.001	18.00 (10.90)	19.00 (12.00)	<0.001
AST (U/L)	22.00 (8.30)	22.00 (9.90)	0.797	22.00 (8.30)	22.30 (9.50)	0.034
TBil (μmol/L)	12.80 (6.80)	13.10 (6.20)	0.131	12.80 (6.90)	12.90 (6.50)	0.931
Scr (μmol/L)	77.50 (29.00)	76.90 (29.00)	0.107	78.00 (28.00)	76.00 (29.00)	<0.001
BUN (mmol/L)	5.80 (2.26)	5.70 (2.05)	0.002	5.83 (2.29)	5.67 (2.07)	<0.001
SUA (μmol/L)	332.93 ± 99.46	347.54 ± 95.61	<0.001	333.43 ± 99.44	344.32 ± 97.40	<0.001

Data are shown as means ± standard deviation for normally distributed variables, median (interquartile range) for non-normally distributed variables, and percentages for categorical variables. DM: Diabetes mellitus; BMI: Body mass index; WC: Waist circumference; SBP: Systolic blood pressure; DBP: Diastolic blood pressure; FPG: Fasting plasma glucose; TC: Total cholesterol; TG: Triglyceride; HDL-C: High density lipoprotein cholesterol; LDL-C: Low density lipoprotein cholesterol; ALT: Alanine aminotransferase; AST: Aspartate aminotransferase; TBil: Total bilirubin; Scr: Serum creatinine; BUN: Blood urea nitrogen; SUA: Serum uric acid.

**Table 2:** Performance of four machine learning models for two forecast periods.

Metrics	Machine Learning Models			
	LR	DT	RF	XGBoost
1-year forecast period				
Sensitivity	0.5559	0.5213	0.5824	0.6569
Specificity	0.6876	0.7004	0.6807	0.5972
Accuracy	0.6669	0.6724	0.6653	0.6066
F1 score	0.3432	0.3325	0.3527	0.3433
2-year forecast period				
Sensitivity	0.6232	0.5580	0.5754	0.6130
Specificity	0.6016	0.6612	0.6647	0.6443
Accuracy	0.6078	0.6316	0.6391	0.6353
F1 score	0.4772	0.4653	0.4780	0.4913

LR: Logistic regression; DT: Decision tree; RF: Random forest; XGBoost: Extreme gradient boosting.

## Data Availability

The authors confirm that all data underlying the findings are fully available and can be obtained after submitting a request to the corresponding author.
